# User-Centered Prototype Design of a Health Care Robot for Treating Type 2 Diabetes in the Community Pharmacy: Development and Usability Study

**DOI:** 10.2196/48226

**Published:** 2025-03-18

**Authors:** Ching-Ju Chiu, Lin-Chun Hua, Jung-Hsien Chiang, Chieh-Ying Chou

**Affiliations:** 1 Institute of Gerontology, College of Medicine, National Cheng Kung University, Tainan City, Taiwan; 2 Department of Computer Science and Information Engineering, National Cheng Kung University, Tainan City, Taiwan; 3 Department of Family Medicine, National Cheng Kung University Hospital, College of Medicine, National Cheng Kung University, No. 138, Sheng Li Road, Tainan City, 70403, Taiwan, 886 6-2353535 ext 5210, 886 6-2091433

**Keywords:** robot, diabetes, self-management, middle-aged adult, community pharmacy, older adult, prototype

## Abstract

**Background:**

Technology can be an effective tool for providing health services and disease self-management, especially in diabetes care. Technology tools for disease self-management include health-related applications for computers and smartphones as well as the use of robots. To provide a more effective continuity of care and to better understand and facilitate disease management in middle-aged and older adult patients with diabetes, robots can be used to improve the quality of care and supplement community health resources, such as community pharmacies.

**Objective:**

The aim of this study was to develop a health care robot prototype that can be integrated into current community pharmacies.

**Methods:**

Three user-centered approaches were used: (1) review of the literature on technology use among older adults, 2) reference to the seven key diabetes self-care behaviors by the American Association of Diabetes Educators (AADE), and (3) meeting with health care providers in the community. Field investigations and interviews were conducted at community pharmacies and diabetes health education centers to determine the appearance, interface, content, and function of the robot.

**Results:**

The results show that diabetes health care prototype robots can be established through user-centered design. The following important features were revealed: (1) perceived ease of use is considered a friendly operating interface; therefore, we used less than 3 buttons in an interface; (2) minimization of the interface between blue and yellow, which is unfriendly to older adults; (3) the health education mode was the most preferred mode with sound, image, and video presentation; (4) the most predilected functions are health education resources and health records, and that patient data can be easily collected through health education games and dialogue with robots; and (5) touching the screen is the most preferred operation mode.

**Conclusions:**

An evidence-based health care robot can be developed through user-centered design, an approach in which a model that connects medical needs to people with health conditions can be built, thereby facilitating the sustainable development of technology in the diabetes care field.

## Introduction

Diabetes is a significant global health issue, with the number of people affected by the disease continually rising [1]. According to the International Diabetes Federation Diabetes Atlas, the global number of people with diabetes reached 537 million in 2021 and is expected to continue rising, reaching an estimated 783 million by 2045 [2]. The most prevalent form of diabetes is type 2 diabetes, which affects more than 90% of all individuals with diabetes [2]. Uncontrolled diabetes over time can lead to severe complications, including cardiovascular diseases, lower-limb amputation, kidney damage (nephropathy), nerve damage (neuropathy), and blindness (retinopathy) [2,3]. Age is one of the risk factors for type 2 diabetes, with its prevalence and mortality rates rising as individuals get older [4,5]. According to statistics from Taiwan’s Ministry of Health and Welfare, the prevalence of diabetes increases significantly from the age of 45 years for both men and women [6]. As the duration of diabetes and patient age increase, patients with diabetes face not only a higher incidence of complications but also challenges related to multiple medications and comorbidities [7]. This underscores the importance of focused health care management for middle-aged and older adult patients with diabetes.

In recent years, technology has emerged as a crucial tool for disease management, including diabetes [8,9]. The demand for digital health care has surged, evidenced by the introduction of more than 90,000 new health-related apps on platforms like the Apple Store and Google Play in 2020 alone, contributing to a total of over 350,000 health care applications available globally [10]. Among these technological advancements in health applications, health care robots are one of the future trends in health technology [11], driven by the ongoing digitization of health care services.

Community pharmacies play a vital role in providing health care services, offering convenient access to medications and health services that support local health care needs. There are currently more than 7400 health insurance pharmacies across Taiwan [12], and a 2016 survey indicated that over 80% of these pharmacies are open for at least 12 hours, with around 40% operating almost year-round [13]. Compared to hospitals, community pharmacies face less pressure from time constraints and space limitations, positioning them as key players in bridging the gap between individuals and larger medical institutions.

Despite the availability of professional medical information in clinical settings, there remains a challenge in ensuring that patients effectively absorb and apply health education and professional advice once they return to their communities. This gap highlights the need for technological tools that support continuous care, especially for middle-aged and older adults managing diabetes. Integrating robots into community health resources, such as community pharmacies, could enhance the quality of care, improve health management, and advance health promotion within the community. The expertise of pharmacists, coupled with the high accessibility of community pharmacies, makes them an ideal setting for such interventions.

However, current technology development for disease management often excludes medical professionals from the design process and lacks real-world testing [14]. Additionally, there is no clear evidence that technology can effectively integrate with community medical resources or bridge the gap between medical institutions and personal care. Therefore, the aim of this study was to design and develop a prototype of a diabetes care robot that meets the needs of middle-aged and older adult patients with diabetes, aligns with the perspective of community pharmacist care, and can be integrated into the current community pharmacy setting.

## Methods

### Participants

The design and development of the prototype robot in this study were primarily conducted through literature-based theories and expert team meetings [15]. The development of the prototype robot involved the researcher synthesizing the literature and conducting field visits to pharmacies and health education units. The development goals and direction were then jointly decided during team meetings. The development of the prototype robot began in March 2018. The team included 1 expert in technology, 1 expert in gerontology, 2 physicians, 12 to 15 engineers and research assistants, and 1 researcher. To maintain progress and continuously refine the prototype robot, the team held regular meetings 1‐2 times per month until a more complete interactive functionality was established to enhance the user experience. Furthermore, to gain a more realistic understanding of the feasibility and initial results of the robot’s application in community health care, 30 middle-aged and older adult patients with diabetes, along with 10 community pharmacists, were also included to provide user feedback.

### Ethical Considerations

This study was performed in accordance with the relevant guidelines and regulations, including the Declaration of Helsinki and was approved by the Institution Review Board (IRB) of National Cheng Kung University Hospital in Taiwan (No. A-ER-105‐509). All the participants provided signed informed consent, and all study data were deidentified to protect the identity of participants. Participants did not receive any financial or material compensation for their participation in this study.

### Procedures

**Figure 1.**
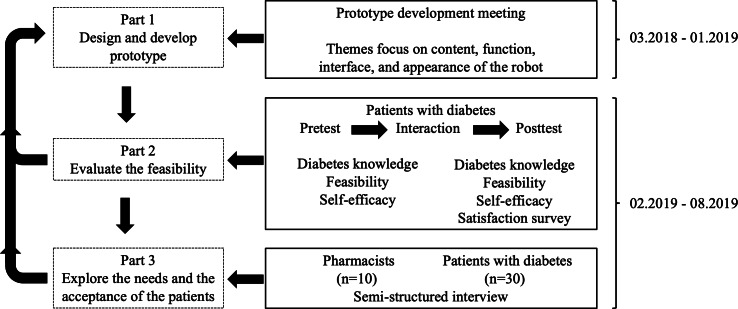
The research design framework for the prototype robot used in community-based diabetes care.

The research design framework for the prototype robot comprised three steps: using an evidence-based literature review and team meeting to design and develop the robot prototype, evaluating feasibility through collecting pre- and postassessment questionnaires, and exploring needs and acceptance through interviews with the target population, as illustrated in Figure 1.

**Figure 2.**
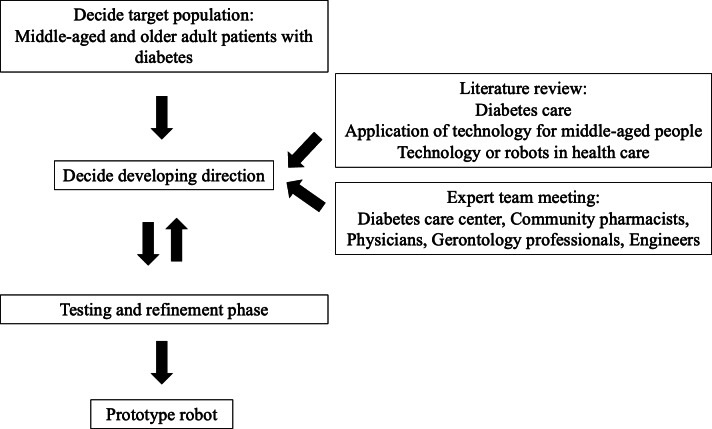
Process showing how to build a prototype community-based diabetes care robot.

Although robotics is an emerging technology, there is a lack of understanding about the applications of robotics. To gain a better understanding of user needs, a high-fidelity prototype can be used to provide users with real-world experience and stimulate their needs, thus enabling them to provide more in-depth insights for future development and design [16]. Therefore, the first part of the study was to design and build a prototype community-based diabetes care robot to facilitate user experience, as illustrated in the detailed flowchart shown in Figure 2.

The second and third parts were dedicated to testing and evaluating the prototype robot. In the second part, it took 15‐20 min for the patients with diabetes to interact with the robot. Before and after the interaction, questionnaires including a diabetes knowledge test, self-efficacy for diabetes, and feasibility of use of the robot were administered. In the third section, semistructured qualitative interviews were conducted with middle-aged and older adult patients with diabetes and community pharmacists about their experiences and visions for using the robots. The detailed methods and results of the pre- and postassessment questionnaires, including a diabetes knowledge test, self-efficacy for diabetes, and the feasibility of using the robot in patients with diabetes, as well as the in-depth interviews with both pharmacists and patients with diabetes, were addressed in our previously published study [17]. This prior study demonstrated that robot interaction significantly improved patients’ diabetes knowledge and the perceived feasibility of its use in pharmacies. Both patients and pharmacists gave positive feedback, with patients valuing self-directed learning, comfortable interaction, and vivid engagement, while pharmacists highlighted real-world applicability and new service potential. These findings offer foundational insights that inform the design and objectives of our current research.

### Design and Construction of a Prototype Community-Based Diabetes Care Robot

**Figure 3.**
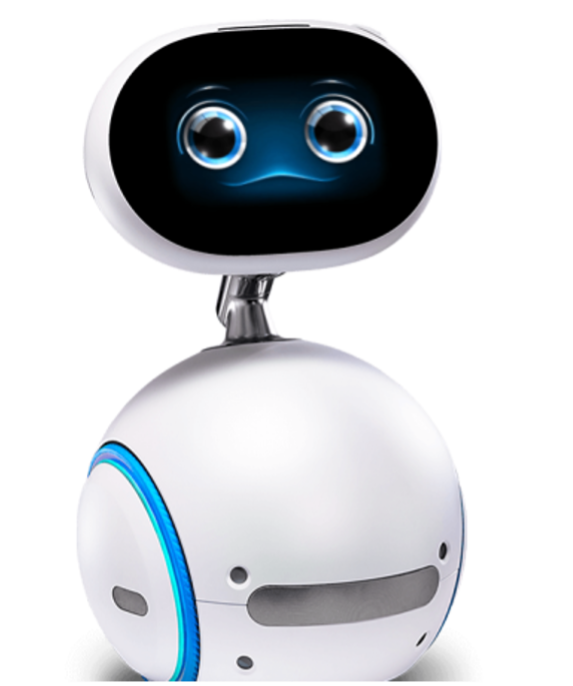
Appearance of the Zenbo robot.

As the goal of this research is to develop a prototype for a community-based diabetes care robot and to gather and understand the perspectives and needs of patients with diabetes and community health care providers, the initial team meetings decided to use an existing robot with foundational functions. This robot was optimized and gradually modified to develop interactive features relevant to diabetes care, aiming for efficiency and smooth user operation. The robot developed in this study uses Zenbo (ASUS), a smart home product from ASUS. The appearance and specifications are shown in Figure 3 and Textbox 1, respectively.

Textbox 1. **Zenbo robot specifications and functions; source: ASUS.**

Specifications:

- External dimensions: 37 × 37 × 62 cm (length × width × height)
- Weight: 10 kg
- Operating system: Android
- Display screen: 10.1" LCD screen
- Language skills: Chinese
- Functions: Listening, speaking, seeing, emotional expression, playing music and videos, movement, internet connection, and learning

LCD: liquid crystal display

The concept for the prototype robot is primarily derived from research and theory, which must align with the needs of the target population, namely middle-aged and older adult patients with diabetes. Therefore, this study focused on theories and care indicators related to diabetes care [18], collected and organized the design and limitations of technology applications for middle-aged individuals [19,20], and examined the development and application of current technology or robots in health care, as well as the connection between the use of technology and health in middle-aged people and the mode for delivering health services [21], to establish the development objectives [22,23]. Three user-centered approaches were used in the design and construction of a prototype community-based diabetes care robot: (1) a review of the literature on technology use among older adults, (2) reference to the seven key diabetes self-care behaviors by the American Association of Diabetes Educators (AADE), and (3) meetings with health care providers in the community. The literature review search strategy of this study involved collecting studies related to the application of robots in the elderly population [24-27]. Field investigations and interviews were conducted at community pharmacies and diabetes health education centers to determine the design of the robot. Because patients with diabetes go to community pharmacies to take their medications, and in addition to providing pharmaceutical services, community pharmacies can also offer related information on disease care. Therefore, in addition to collecting service experiences from the community pharmacists, the choice of a community pharmacy as the testing site for the prototype is driven by the fact that these pharmacies provide a secure, private, and professionally consultative environment.

The design framework of the community-based diabetes care robot was devised to collect and compile data in four main areas: appearance, interface, content, and function. For appearance, we collected data about the appearance and size of the robot. For interface, data were collected about the color, text size, arrangement, and other screen design. For content, the system was responsible for building the information that needed to be conveyed, with the user’s needs as the primary consideration. For function, we collected data about the presentation of the interactive process and information transfer.

Once the direction of development has been established, a prototype robot is initially built that can be used in practice. The development of technology does not only rely on past and proven experience, but also must be tailored to the needs of real users. Therefore, the prototypes need to interact with the users in real life and give them a real experience in accordance with the proposed development directions. It is only after this that users can give practical advice.

## Results

Using a literature search and practical experience, this study focused on data collection in four main areas: appearance, interface, content, and function of robot design. The development directions and options for prototype robots are listed in Table 1.

**Table 1.** Development directions and options for prototype robots.

	Literature and theory	Interviews and team discussions	Results and design directions
Appearance	Type: The humanoid type is less well accepted. The doll type, on the other hand, is seen as a toy for children [22]. Prototype robots should avoid being too complex or trendy. However, if the prototype lacks a humanoid feel, it will not be favored by senior citizens [23]. Application mode: The focus of the robot varies between different appearances. Appearance will affect whether it is presented in a functional or interactive way [28,29].	Size: The prototype robot must fit into the space of the pharmacy. Height: It should be at a height at which middle-aged to older adults can sit and see it horizontally.	It must be confirmed that this model is not complicated and is expected to be well-accepted because it is a non-humanoid model. However, it may be seen as a toy because of the cute non-humanoid appearance. The model does not have hands and feet to operate and therefore operates mainly in a conversational manner. It must be confirmed that the angle and height of the interaction mode (eg, the height of the table or chair on which the robot can be placed) can be adjusted.
Interface	Colors: Blue and yellow are less recognizable [19]. Yellow and green combinations, and red and purple combinations, are prone to recognition errors [20]. Operation: Per the Technology and Acceptance Model [30], the interface and operation must be simple because it is used by older adults. The font should be large and clear. The interface should have a maximum of four buttons. All other interfaces have two buttons for simple selection [31].	The interface should be simple. A single screen does not need much emphasis. Text should be enlarged.	The color scheme of the interface is highly contrasting to avoid confusion over similar color schemes. The use of blue and yellow colors is reduced and the number of buttons on the interface is 2‐3. The content is centered, and apart from the main screen, the content is mostly presented as a single theme. The font size ranges from a minimum of 30sp to a maximum of 50sp (sp being the text unit in the Android system).
Content	Seven key diabetes self-care behaviors by the American Association of Diabetes Educators [18]	Applications addressing various everyday challenges and health education videos on common diseases in community pharmacies.	The content of the system is based on the seven diabetes self-care behaviors, with games and interactive dialogues to present a variety of health education methods. To make the content more relevant to the public, common disease symptoms and complications of diabetes are presented.
Function	Operation: Technology and Acceptance Model [30]. Health education: Multisensory perception stimulates learning [21], with a core health education focus each time. Technology applications: The applications of robots in health management can include disease management, monitoring of physiological data, and interactive health education [26,32,33]. The use of technology in diabetes health management focuses on recording and reminding, as well as providing information and entertainment [34-36].	There are games, friendly greetings, and flexible interaction times.	The health education mode is presented with sound, images, and video, avoiding a focus on the use of a single sense. The functionality of the current model will be considered and the information on functionality available in the literature will be further researched.

### Appearance

Although the robot’s appearance was determined based on the available resources, the chosen model features a cute, non-humanoid design to better align with the preferences of older adults and enhance user acceptance. Although the robot’s height is a limiting factor, placing the robot on elevated surfaces, such as tables or chairs, can correct height-related issues, making it easier for users to operate.

### Interface

As the interface is critical to the overall smoothness of the robot, the presentation of the interactive interface must meet the needs of middle-aged and older adults in terms of the ease of use of technology products and text reading as much as possible. In addition, according to the Technology and Acceptance Model [30], the perceived ease of use should be considered as a whole and should take into account the user-friendliness of the interface so that the user can experience the clearest and easiest process.

In the interface development, to make it easiest for older adults to operate, each screen will display a maximum of two main options, with options clearly framed to reduce the likelihood of users being unsure of their choices. Information and selection buttons will be centrally placed and maximized on the screen to help users quickly focus on key points. The main screen will feature simple text and image displays. To avoid causing difficulties in recognition for older users, the background will be a single color, and the overall interface will use no more than two main color tones. The main screen and option areas will predominantly use a black-on-white color scheme, with high contrast to make text easier to read.

### Content

The main content of the development includes the seven key diabetes self-care behaviors according to the AADE standards. The overall main content and functions presented by the prototype robot are detailed in Table 2 and aligned with the seven AADE diabetes care indicators.

The main functions of the system are health education resources and health records. Health education resources are presented through health education quizzes, pamphlets, and videos. The health records section includes health values, discomfort, and nutrition. The concept of this record is to help patients with diabetes manage their records, including basic physiological data such as blood pressure, blood glucose, and blood lipids. The relevant data are entered and the corresponding standard values are provided. The focus of the “Discomfort” program is to understand and record the various conditions in the patients with diabetes and to compare the complications associated with diabetes with the common problems of the family. By reminding and advising patients to be more alert to their health conditions, they can gain a better understanding of their health conditions. The results are recorded for follow-up. All interactions can be fed back to the user in the form of paper or communication software messages.

**Table 2.** Functional development and presentation of prototype robots.

Theme	Subtheme	Theme content	Presented at the American Association of Diabetes Educators
Health education resources	Health education test	Questions focus on the seven major areas of diabetes care and common diabetes health problems	Healthy eating, regular exercise, blood glucose monitoring, proper medication use, problem solving, health adaptation, and risk reduction
	Health education leaflet	Share and recommend links to help filter relevant content	Healthy eating, regular exercise, blood glucose monitoring, proper medication use, problem solving, health adaptation, and risk reduction
	Health education videos	Share and recommend links to help filter relevant content	Healthy eating, regular exercise, blood glucose monitoring, proper medication use, problem solving, health adaptation, and risk reduction
Health records	Health values	Alert and record blood pressure, blood glucose and lipids, and provide standard values	Blood glucose monitoring
	Discomfort	Presented in a lifestyle format to remind patients of complications and other problems they may encounter and link them to their disease conditions	Problem solving, health adaptations, and risk reduction
	Nutrition	Ask each other about their dietary status in a simple conversation to help them develop a balanced diet	Healthy eating

### Function

To establish the overall function, we should first understand the current mode of application of technology in disease and health management, including the application of health technology for older adults, diabetes management technology tools, and the application of robots in health management, and consider the current operation fluency and feasibility of using robots. According to the current functional status of the robot, the main functions are primarily simple question-and-answer interactions with pictures and images, so the main functions are presented in a health education manner. A game was used to transfer knowledge on diabetes-related care content, and a thematic health education video was shown as feedback to the patients according to their wrong answers.

The main objective of the development of this prototype robot is to enable patients with diabetes to experience actual interaction with the robot and to understand the possible interaction patterns, in order to understand the experience and perspective of the user. During the development process, the prototype robot is adapted to meet the needs of the community and medical personnel based on the health education needs of the Diabetes Health Centre and the feasible applications of the community pharmacy. In view of the time and cost of development as well as the operation of the technology, the functions were not expanded extensively and were mainly presented in the form of a basic framework. After the patient with diabetes or community health care worker has confirmed the suitability of the directions and content, the functions will be expanded and built. To allow each participant to experience the same features, the most stable and fully developed part (ie, the health education test, a game with the robot, that focuses on diabetes-related knowledge) is used as the main axis for participant interaction. The rest of the developed functions are presented as introductions and simple demonstrations.

## Discussion

### Principal Findings

This study demonstrated that an evidence-based health care robot can be developed through user-centered design, facilitating the sustainable advancement of technology in the field of diabetes care. The use of technology in patient management can be beneficial to patients. However, the applications of technology that are readily available on the market are not always effective. While the current technology interventions are mostly aimed at behavioral change, they are rarely supported by therapeutic guidelines and theories [35,37], making the development process too subjective or deviating from reality. The core elements of technology development must therefore be user-centered. Thus, based on the technology being developed and designed for diabetes disease self-management, Goyal et al proposed four stages of user-centered development [38]. The first is the so-called development process, which must be evidence-based and analyze possible barriers and problems and must be culturally and environmentally appropriate. In the second stage, the feasibility of the system must be understood. The third stage involves further evaluation of effectiveness. The fourth stage requires more long-term monitoring. At the same time, as the four stages are in a continuous cycle and interact with each other, it is necessary to revise and review them repeatedly to build a technology tool that meets the needs of users [38]. Furthermore, personal health information must also be protected. While this prototype requires login, ongoing efforts should be made to enhance and ensure information security.

### Comparison With Prior Work

In previous studies of diabetes care robots, the main focus has been on younger age groups of patients with type 1 diabetes [32,33,39], and less on middle-aged patients with type 2 diabetes. Even in the case of older adult care, the functional applications of robots are mainly companionship-based or focus on social functions for older adults. There should be less involvement in behavioral change, but this is also an important part of health messaging. Therefore, this study uses the seven key diabetes self-care behaviors by the AADE to consider the basics of healthy eating, regular exercise, blood glucose monitoring, and proper medication use as core health-enhancing skills, and to help patients with diabetes better understand their disease and their health. These seven self-care behaviors are interlinked with diabetes disease self-management and not only directly contribute to disease management (eg, reminders and records), but also enhance the overall quality of care for the disease by building up the correct concepts of patients with diabetes. This content is presented as a basic health education leaflet and a health education video to convey information. The health education resources are sourced from relevant health education websites and reference links from major hospitals and institutes, and can be categorized to help patients quickly search and link to relevant functions. In terms of the functions, we should first understand the current mode of application of technology in disease and health management, including the application of health technology for older adults, diabetes management technology tools, and the application of robots in health management, and consider the current operation fluency and feasibility of using robots. The robot itself has basic functions such as playing videos and documents. Based on the current functionality of the robot, the main focus of further development is on the simple interaction with pictures and images. The main function is therefore presented in the form of health education. To make the health education interaction more efficient and interesting, a health education game was used to transfer knowledge about diabetes-related care content, and at the end of one stage of the game, a health education video on the relevant topic was selected and shown as feedback based on the questions that the patient answered incorrectly, to deepen the patient’s understanding of the concept.

Past studies have also supported the idea that a multimedia approach to health education can help patients understand the content of health education [40,41]. The health education model is more stimulating, as it delivers information to patients in a variety of ways, such as videos and animations, which in turn produces better health education results. This is one of the main advantages of robots for the implementation of health education [40]. The robot’s rich expressions and gestures, as well as its voice-to-voice interactions, are an advantage in teaching [41]. The prototype utilized in this study is a nonhumanoid robot. While humanoid robot interactions are more human-like, their realistic appearance can often induce fear, leading to lower acceptance. In contrast, nonhumanoid robots, though cute, might be perceived more like toys, resembling a game. The advantage lies in a more engaging interaction, but the downside is that they may be seen merely as playthings, potentially overshadowing the educational information they convey.

In the delivery of health services by robots, there is a need for a thorough consideration of the technological side, the professional side, and the patient side. Therefore, in addition to the professional development in the field of technology, the initial development should also identify and create a suitable development direction from the perspective of medical professionals, and build a prototype robot. Later on, the prototype robot can be used to enable patients with diabetes to experience the interaction with the robot, and through this interaction, to gather more in-depth information on patients’ thoughts and experiences on the application of technology. In the context of the rapid expansion of technology, it is important to have more detailed information on the user experience so that subsequent developers can have a clearer direction, and at the same time, have the most relevant technological tools for the core users (ie, older adult patients).

### Strengths

The main advantage of the robot developed in this study is that the prototype was developed using data from professional team meetings, literature, real-world data, and experience interviews, as well as the participation of people from the relevant professional fields. This comprehensive approach ensured the prototype is robust. In addition, the development of the prototype robot was not a one-off exercise. The aim was to create a prototype that would enable actual interaction with patients with diabetes, not just from the developer’s perspective. Each time further progress is made, it will be compared and revised with clinical practitioners to create a more realistic diabetes care robot that can actually interact with patients with diabetes in the real world.

### Limitations

The literature reviewed in this study primarily comes from Western countries, which may not fully correspond to the disease characteristics of East Asian populations. Differences in illness, age, and cultural backgrounds may affect users’ perspectives. To mitigate this limitation, future studies could be conducted in Western countries to validate the findings and evaluate their applicability in different contexts. In addition, the testing site for this study was a community pharmacy in an urban area, so it does not provide insights into how elderly individuals with different lifestyles or from different regions perceive and use the robot. Consequently, the findings may not be fully applicable to all health care settings. Future research could extend to rural community pharmacies or other community health care settings, including community hospitals, clinics, and chain pharmacies, to test the application of health care robots. Moreover, the size and functionality of the robots used in this study, along with the critical role of the internet in their operation, may limit their capabilities. For example, uninterrupted and smooth conversations rely on a stable network connection. Interruptions or network issues can hinder the robot’s performance. Efforts were made to ensure a stable network environment during testing, but future studies should consider testing in various network conditions to better understand the impact of connectivity on robot functionality.

### Conclusion

The prototype diabetes care robot was developed using data from the literature, professional team meetings, and practical interviews, with the aim of creating a more evidence-based health care robot that can actually give patients with diabetes a realistic experience for their disease care and enable them to give clearer and more specific feedback for the subsequent development and application of technology in disease care.

## Abbreviations

AADE: American Association of Diabetes Educators
